# Performance of Layer-by-Layer-Modified Multibore^®^ Ultrafiltration Capillary Membranes for Salt Retention and Removal of Antibiotic Resistance Genes

**DOI:** 10.3390/membranes10120398

**Published:** 2020-12-06

**Authors:** Robert Niestroj-Pahl, Lara Stelmaszyk, Ibrahim M. A. ElSherbiny, Hussein Abuelgasim, Michaela Krug, Christian Staaks, Greta Birkholz, Harald Horn, Tian Li, Bingzhi Dong, Lars Dähne, Andreas Tiehm, Stefan Panglisch

**Affiliations:** 1 Surflay Nanotec GmbH, Max-Planck-Str. 3, 12489 Berlin, Germany; g.birkholz@surflay.com (G.B.); l.daehne@surflay.com (L.D.); 2 Technologiezentrum Wasser, Karlsruher Straße 84, 76139 Karlsruhe, Germany; lara.stelmaszyk@tzw.de (L.S.); andreas.tiehm@tzw.de (A.T.); 3 Lehrstuhl für mechanische Verfahrenstechnik/Wassertechnik, Universität Duisburg-Essen, Lotharstrasse 1, 47057 Duisburg, Germany; ibrahim.elsherbiny@uni-due.de (I.M.A.E.); hussein.abuelgasim@uni-due.de (H.A.); stefan.panglisch@uni-due.de (S.P.); 4 inge GmbH-DuPont, Flurstraße 27, 86926 Greifenberg, Germany; mkrug@dupont.com (M.K.); christian.staaks@dupont.com (C.S.); 5 Karlsruher Institut für Technologie, Engler-Bunte Institute, Wasserchemie und Wassertechnologie, 76131 Karlsruhe, Germany; harald.horn@kit.edu; 6 College of Environmental Science and Engineering, Tongji University, Shanghai 200092, China; litian001@tongji.edu.cn (T.L.); dongbingzhi77@126.com (B.D.)

**Keywords:** layer-by-layer technique, polymeric membranes, antibiotic resistance genes, salt retention, membrane cleaning

## Abstract

Polyether sulfone Multibore^®^ ultrafiltration membranes were modified using polyelectrolyte multilayers via the layer-by-layer (LbL) technique in order to increase their rejection capabilities towards salts and antibiotic resistance genes. The modified capillary membranes were characterized to exhibit a molecular weight cut-off (at 90% rejection) of 384 Da. The zeta-potential at pH 7 was −40 mV. Laboratory tests using single-fiber modified membrane modules were performed to evaluate the removal of antibiotic resistance genes; the LbL-coated membranes were able to completely retain DNA fragments from 90 to 1500 nt in length. Furthermore, the pure water permeability and the retention of single inorganic salts, MgSO_4_, CaCl_2_ and NaCl, were measured using a mini-plant testing unit. The modified membranes had a retention of 80% toward MgSO_4_ and CaCl_2_ salts, and 23% in case of NaCl. The modified membranes were also found to be stable against mechanical backwashing (up to 80 LMH) and chemical regeneration (in acidic conditions and basic/oxidizing conditions).

## 1. Introduction

Layer-by-layer (LbL)-coated membranes are gaining research interest not only for water filtration application, but also for recycling of waste solutions [1,2], reverse osmosis (RO) [3] and desalination processes [4]. LbL-coated membranes use the advantage of a high water flux, because of ultrafiltration membrane support, as well as increased filtration ability due to the LbL polyelectrolyte coating. The retention of ions and other charged substances is substantially influenced by the surface charge of the coating. The simplest way to alter the surface charge is to modify the outer most layer of the LbL coating to polycation or polyanion. Besides, the salt and antibiotic resistance genes’ (ARGs) retention are influenced by the number of bilayers, the ionic strength of the background solutions and/or the type of polyelectrolytes [5]. The polyelectrolyte coating also should be stable toward chlorine regeneration. In this study, the employed strong polyelectrolytes, polydiallyldimethylammonium chloride (PDADMAC) and polystyrenesulfonate (PSS), are pH-independent and resistant to certain amounts of chlorine [6,7].

In addition to the high flux of the ultrafiltration membranes, the use of capillary membranes has the advantage of a lumen side separation layer (inside out filtration), hence the crossflow velocity can be precisely controlled. Furthermore, the modules can be regenerated by backwashing and the ready-to-use (i.e., already assembled) modules can be modified using the layer-by-layer technique.

Due to the use of large quantities of antibiotics in human, veterinary medicine and animal husbandry (including factory farming for food production and aquaculture), antibiotic-resistant bacteria and antibiotic resistance genes (ARGs) have increased significantly in the clinical area in recent years. ARGs can enter the environment through the agricultural use of sludge from livestock farms and fermentation residues, or the discharge of sewage treatment plant effluents from municipal wastewater [8,9]. Due to the rapid spread and transfer of resistance genes in the environment [10,11,12,13,14], ARGs were detected in several surface water and drinking water treatment plants worldwide [11,15,16,17,18]. Conventional treatment strategies are designed to eliminate the major organic contaminants and hygiene-relevant microorganisms from drinking water [19]; however, they are mostly insufficient to eliminate residual ARGs [20]. Moreover, ARGs can exist in environmental bacteria [18] and as extracellularly bound or unbound DNA [21]; thus, they can reach the end of a treatment chain and be released into the water networks. The incomplete elimination of ARGs during water treatment poses a certain risk for human health since horizontal gene transfer processes might contribute to further distribution of antibiotic resistance among various bacterial species [22]. Different approaches for removing ARGs from water are currently used in wastewater treatment plants [23,24,25,26]. Membrane filtration is a worldwide emerging method for drinking water preparation [27,28,29,30]. The application of ultra-, micro- and nano-filtration membranes can contribute to reduce the ARG content in drinking water [31].

In the current study, polyether sulfone (PES) Multibore^®^ ultrafiltration membranes were modified using the layer-by-layer technique and employing polydiallyldimethylammonium chloride and poly-(4-styrenesulfonic acid). The modified membrane modules were characterized via zeta-potential and molecular weight cut-off (MWCO) measurements. In addition, the pure water permeability and single salt retention were determined using a mini-plant membrane testing unit. The ARG retention was evaluated in lab-scale experiments employing specially modified single-fiber membrane modules. Furthermore, the stability of the polyelectrolyte coating was tested via applying a range of different backwashing fluxes; thereafter the retention of bivalent salts was measured.

## 2. Materials and Methods

### 2.1. Membrane and Chemicals

The polyether sulfone (PES) ultrafiltration membrane modules, comprising 10 Multibore^®^ fibers, each containing 7 capillaries with an inner diameter of 0.9 mm, were supplied by inge GmbH (DuPont, Greifenberg, Germany). Each membrane module has an overall membrane surface of 0.05 m^2^ and molecular weight cut-off (MWCO) of 100 kDa. Polydiallyldimethylammonium chloride (PDADMAC) (20% solution with a molecular weight of 250–350 kDa) and poly-(4-styrenesulfonic acid) (PSS) (molecular weight of 1000 kDa) were purchased from Sigma Aldrich (Schnelldorf, Germany). NaCl, MgSO_4_, CaCl_2_ and NaOCl were purchased from Carl Roth GmbH (Karlsruhe, Germany). All salts were of analytical grade.

### 2.2. Semi-Automated LbL-Coating of Ultrafiltration Membranes

**Figure 1:**
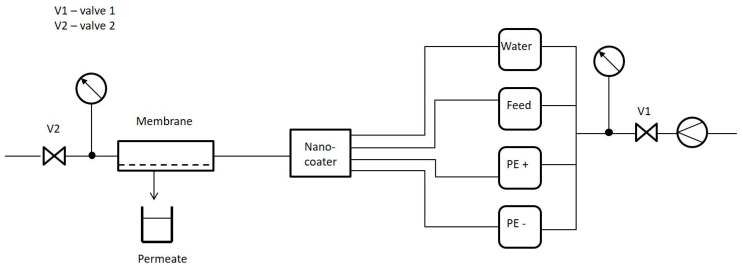
Schematic illustration of the “NanoCoater” system used for coating the capillary membranes using polyelectrolytes. PE = polyelectrolyte solutions.

For the semi-automated coating process of the Multibore^®^ ultrafiltration membrane modules, Surflay developed a device named “NanoCoater”. A schematic illustration is shown in Figure 1. It allows precise and timer-controlled coating. Polyelectrolyte solutions and ultrapure water for washing were supplied for the coating device. The membrane was connected to the output valve of the NanoCoater. The coating solutions and water were introduced by timer-controlled valves into the membrane under pressure. For controls and programming, a Windows^®^-based Virtual Basic (VBA) program was used. The membranes were coated dynamically, which means that the polyelectrolyte solutions are filtered through the membrane during the coating.

#### Coating Procedure

PDADMAC and PSS solutions were prepared at a concentration of 1 g∙L^−1^ in 0.1 M NaCl. According to Maroni et al., around 0.5 mg of PDADMAC or PSS can coat 1 m^2^ of membrane area [32]. The polyelectrolyte reservoir coupled to the NanoCoater contains 1 L, so it is capable of coating around 2000 m^2^ of membrane area. One membrane module has an overall membrane area of 0.05 m^2^, while a single Multibore^®^ fiber has a membrane area of 0.005 m^2^ (25 cm length).

The polycation was first introduced into the system; Valve 2 (Figure 1) was closed, so the polyelectrolyte solution was filtered through the membrane at a pressure of 1 bar. Then, the NanoCoater opened Valve 2 and flushed out the remaining solution by pressurized air. Then, the capillaries were flushed with ultrapure water to wash out the polyelectrolyte. Again, pressurized air was pumped through in order to remove excess water. Then, the polyanion (PSS) was introduced and the latter process was repeated. The polyanions and polycations were sequentially introduced into the membrane cell. The number of double layers varied according to the filtration approach. The exact number of double layers of PSS and PDADMAC is part of internal research and protected as intellectual property.

Usually, the LbL-coated membranes were used directly after coating for the filtration experiments. In case of long-term storage (more than 1 week), the coated membranes were stored in 15% glycerol. Afterward, the membrane was dried and stored at room temperature until usage.

### 2.3. Coating of PES Microparticles and Zeta-Potential Determination

Zeta-potential measurements were performed employing the PES model microparticles made from PES raw material via solvent precipitation. The microparticles were coated with the same exact polyelectrolyte coating and number of double layers. For the coating, the resuspended and sonicated microparticles were each incubated with a polyelectrolyte solution for 10 min. After coating with a certain polyelectrolyte, the coated microparticles were centrifuged (2000× *g* for 5 min) and washed thrice in ultrapure water. Thereafter, the procedure was repeated using the oppositely charged polyelectrolyte. The zeta potential was measured at pH 7 in 1 mM TRIS buffer via a Zetasizer Nano SP (Malvern Panalytical, Malvern, UK). The zeta-potential was calculated using the Helmholtz–Smoluchowski equation.

The zeta-potential for the pristine PES ultrafiltration flat sheet membrane was measured in 0.1 M KCl for a pH range of 7–10 employing a Malvern Zetasizer (Malvern Panalytical, Malvern, UK).

### 2.4. Evaluation of Performance of the LbL-Modified Membranes

#### 2.4.1. Membrane Filtration Unit

**Figure 2:**
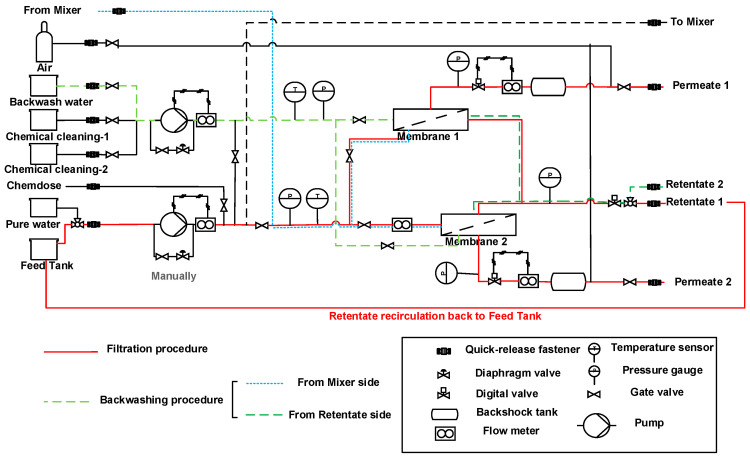
Schematic illustration of the mini-plant membrane testing unit.

Filtration experiments were performed at room temperature (20 °C) via a fully automated mini-plant membrane testing unit, purchased from Convergence (Convergence Industries B.V., Enschede, Netherlands. Mini-plants enable membrane testing at full-scale operating conditions without the need for sophisticated and highly costed pilot-scale constructions [33]. The membrane testing unit can test and control two membrane modules simultaneously using the same feed solution at wide operating pressure values. A schematic illustration is presented in Figure 2. The filtration and cleaning procedures were programmed. The values of temperature, density, transmembrane pressure and flow rates were automatically collected every 5 s.

#### 2.4.2. Membrane Pretreatment Prior to Filtration Experiments

Before the filtration experiments, the LbL-modified membranes were cleaned from preservatives by forward flushing of the membrane modules at a crossflow velocity of 0.6 m·s^−1^ for 15 min followed by a membrane compaction step using ultrapure water (conductivity 2–5 µS·cm^−1^, total organic content < 0.2 mg·L^−1^) with recirculation of the retentate at the same crossflow velocity for 4 h.

#### 2.4.3. Measurement of Pure Water Permeability and Salt Retention

Pure water permeability was determined by measuring the membrane flux at a crossflow velocity of 0.22 m·s^−1^ and transmembrane pressure of 2.5 bar for 15 min. Pure water permeability was determined 3 times using Equation (1), and the average values were reported.

(1) $$Permeability\;\left(W\right)=\frac{J}{TMP}$$

where *W* is the membrane permeability (L·m^−2^·h^−1^·bar^−1^); *J* is the water flux (L·m^−2·^h^−1^); and *TMP* is the transmembrane pressure (bar).

Salt retention experiments were performed in the form of two filtration cycles, each lasting for 1 h using MgSO_4_, CaCl_2_ and NaCl single salts at a concentration of 600 mg·L^−1^, crossflow velocity of 0.22 m·s^−1^ and transmembrane pressure of 2.5 bar. After each filtration cycle, forward flushing at a crossflow velocity of 0.6 m·s^−1^ for 5 min was applied. Normalized permeability was determined and plotted during the two filtration cycles using Equation (2)

(2) $$Normalized\;permeability\;\left(W^{\prime }\right)=\frac{W_{t}}{W_{i}}$$

where $W^{\prime }$ is the normalized permeability; $W_{t}$ is the permeability measured at filtration time *t*; and $W_{i}$ is the initial permeability that is pure water permeability. Permeate samples were collected in each filtration cycle at different time intervals from the starting of the experiments: 10, 30 and 50 min for 10 min. Membrane retention was calculated via Equation (3)

(3) $$Retention\;\left(\%\right)=1-\left(\frac{C_{P_{t}}}{C_{F_{i}}}\right)\cdot 100$$

where $C_{P_{t}}\;\mathrm{is}\;$solute concentration in the permeate at filtration time *t*, measured in terms of conductivity; and $C_{F_{i}}$ is the solute concentration in the actual feed solution, measured in terms of conductivity. Conductivity was measured through a high-performance laboratory conductivity meter, inoLab@Cond 7310 (Xylem Analytics Germany Sales GmbH & Co. KG, WTW, Weilheim, Germany).

#### 2.4.4. Measurement of Molecular Weight Cut-Off

The modified membrane modules were characterized regarding their MWCO, employing polyethylene glycol (PEG) with a molecular weight range of 62–2000 Da, purchased from Sigma-Aldrich—St.-Louis, MO, USA. A total feed concentration of 2.9 g·L^−1^ was employed, as described in Table 1. Milli-Q water (conductivity 1 µS·cm^−1^, total organic content < 0.2 µg·L^−1^) was used to prepare the PEG feed solution mixture. The portions for different PEG were calculated as double the concentration required from each molecular weight to form a complete monolayer on the membrane surface in case of 100% retention. The particle volume for a certain molecular weight was calculated Equation (4) [34]

(4) $$r=2.792\times 10^{-11}\cdot {\left(Mw\right)}^{0.48}$$

where *r* is the particle radius for the PEG molecule with a certain molecular weight (*Mw*). MWCO tests were performed at a crossflow velocity of 0.6 m·s^−1^, transmembrane pressure of 0.3 bar, pH 6.5 and feed temperature of 23 ± 1 °C for 6 h. Permeate samples were collected after 3.5, 4.5 and 5.5 h. Feed, retentate and permeate samples were preserved in 0.01 mol·L^−1^ sodium azide, ready for analysis. Samples were analyzed using gel permeation chromatography (GPC, Agilent 1200 series).

#### 2.4.5. Investigation of Backwashing Flux Influence on the Stability of LbL-Modified Membranes

The stability of the LbL-modified membranes was investigated via applying different backwashing fluxes over five consecutive filtration cycles. Backwashing (BW) experiments were performed using ultrapure water at four fluxes: 50, 60, 70 and 80 LMH at a maximum BW pressure of 5 bar. Lower fluxes were not applicable due to a limitation of the filtration testing unit. Each BW experiment was performed for 2 min; 1 min from the mixer side and 1 min from the retentate side (cf. Figure 2). The stability of the LbL-modified membranes was investigated via filtration of 600 mg·L^−1^ MgSO₄ solution at a crossflow velocity of 0.22 m·s^−1^ and transmembrane pressure of 2.5 bar for 1 h, and the membrane performance was compared before and after the BW experiments.

### 2.5. Laboratory Filtration Experiments of ARGs

**Figure 3:**
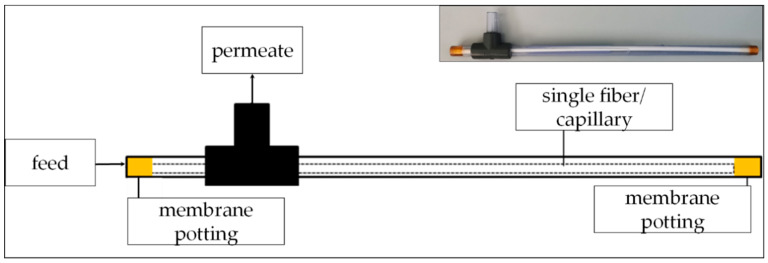
Module containing a single-fiber, modified capillary for the ARG filtration experiments.

For the preparation of the ARGs, autoclaved ultrapure water was spiked with resistance genes of defined lengths. Fragments were amplified with specific primers (shown in Table 2) via PCR and then diluted with nuclease-free water (UltraPureTM Distilled Water, Thermo Fischer Scientific, Dreieich, Germany) to defined concentrations of approximately 10^6^ gene copies∙µL^−1^ in the working solution after fluorometric measurement of the DNA concentration with a Qubit Nanophotometer (Thermo Fischer Scientific, Dreieich, Germany). The listed genes were chosen according to their fragment lengths for the established qPCR reactions and for their relevance in environmental water samples, as confirmed by many previous studies. [11,35,36,37,38]. Before and after the membrane filtration (1 to 5 h-filtration process in total), a feed sample and a sample of the pooled effluent (permeate) were taken and used as the template in the quantitative real-time PCR (qPCR). Additionally, a sample of the retentate was taken after finishing the filtration process. Membrane filtration experiments were performed using specially prepared single-fiber modules with a dead volume of approx. 12 mL, as shown in Figure 3.

To evaluate the effect of the LbL-coating, uncoated and coated PES membrane capillaries were used for the filtrations. The working solution was pumped through the modules using a peristaltic pump. Before operation with the ARG solution, all modules were operated using sterile ultrapure water to rehydrate the dry modules preserved with glycerol and to determine the flow rate. Flow rates for the performed dead-end filtrations differed slightly between the tested capillaries, ranging from 0.9 to 2.2 mL∙min^−1^. All tubing and adapters were previously rinsed with a 500 ppm NaOCl-solution and distilled water.

To determine the efficiency of the gene fragment size-dependent removal of the membranes, the sieving coefficient *S_0_* was determined using Equation (5), according to the work of Ager and Latulippe [45,46].

(5) $$S_{0}=\frac{\mathrm{gc}\left(\mathrm{fil}\right)\;\left[µL^{-1}\right]\cdot \;V\left(\mathrm{perm}\right)\;\left[\mathrm{mL}\right]}{\mathrm{gc}\left(\mathrm{feed}\right)\;\left[{µL}^{-1}\right]\;\cdot \;V\left(\mathrm{feed}\right)\;\left[\mathrm{mL}\right]}$$

where gc(fil) is the gene copy concentration measured in the filtrate; V(perm) is the volume of the permeate fraction (pooled); gc(feed) is the measured gene copy concentration in the feed; and V(feed) is the absolute volume that was used for filtration.

### 2.6. Determination of Interactions Between the ARGs and Membrane

To also determine the ARGs bound to the membrane, a 24 h incubation experiment was performed using coated and uncoated single-fiber capillaries. In detail, 50 mL of the ARG working solution (10^6^ gene copies∙µL^−1^ in ultrapure autoclaved water) was transferred in a sterile 50 mL reaction tube and incubated with 10 cm (cut into 5 pieces of 2 cm, and cut lengthwise) of the membrane at room temperature for 24 h, according to the experiments performed by Latulippe et al. [46]. Before the incubation, the membranes were cut into 5 equally sized pieces of 2 cm and carved lengthwise. After 24 h, total DNA was extracted directly from the membranes by using the Fast DNA^®^ SPIN Kit for Soil (MP Biomedicals) according to manufacturer’s instructions. Therefore, the DNA bound to the membranes was mechanically eluted with Lysing Matrix E Beads in a FastPrep-24 instrument (MP Biomedicals). Additionally, the working solution was sampled at 0 and 24 h, to determine whether there is a reduction of gene fragments in the solution due to adsorption to the membrane. A negative control was run in parallel with a tube containing the ARG working solution without a membrane and for each tested membrane and a tube containing the membrane without the ARG fragments, respectively. Quantification of the gene fragments was performed using quantitative real-time PCR (qPCR).

### 2.7. qPCR Analysis

*16S* ribosomal DNA, the integrase gene, and the resistance genes’ gene copy numbers were determined by quantitative real-time PCR (qPCR) using the same previously published primer sets as shown in Table 2. All qPCRs were performed using a Rotor-Gene 6000 cycler (Corbett) with a SsoFast EvaGreen Supermix (Bio-Rad). The temperature profile for the amplification was as follows: 2 min 98 °C (initial phase for enzyme activation), 45 cycles of 20 s at 98 °C (denaturation), 20 s at a primer-specific annealing temperature (TA), and a fragment length-dependent elongation time (tE) at 72 °C, followed by a melting curve analysis. The TA and tE used are listed for each gene in Table 3.

All samples and standards were analyzed in duplicates. The qPCR standards were prepared from the serial dilutions of known quantities of linearized plasmid-containing target genes. For quality control, the R^2^ of the standard curve as well as the amplification efficiency were determined and melt curve analysis was performed. Only qPCR experiments with R^2^ values > 0.990 and efficiencies between 90 and 105% were considered. Amplification products were verified via QIAxcel^®^ Advanced system (Qiagen GmbH, Hilden, Germany). An overall limit of quantification was 10 gene copies∙µL^−1^.

## 3. Results and Discussion

### 3.1. Characterization of LbL-Modifed Membranes and the Polyelectrolyte Coating

**Figure 4:**
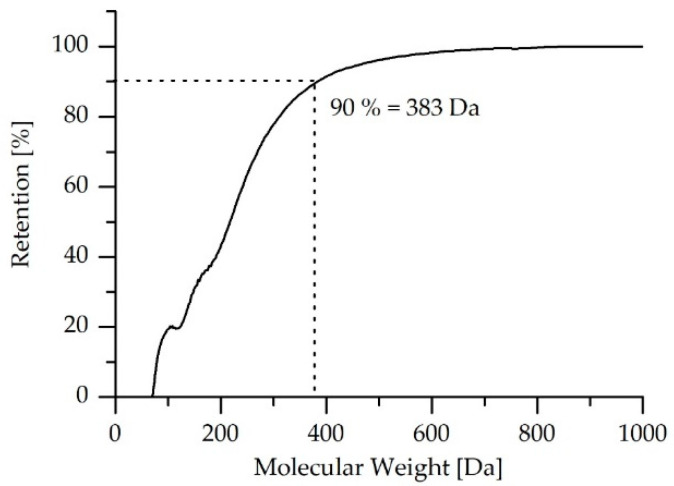
Sieving curve for the LbL-coated PES Multibore^®^ after a 3.5 h filtration duration.

The MWCO for the LbL-coated membranes was measured as described in Section 2.4.4. The zeta-potential for the PEG solution containing different molecular weights revealed that the PEG particles exhibited a negative surface charges with a zeta-potential of −30 mV at the working pH (pH 7), which indicates the stability of the PEG solutions. The sieving curve for the samples collected after 3.5 h, 4.5 h and 5.5 h were plotted to estimate the MWCO at 90% retention. Nevertheless, a representative graph of only permeate collected after 3.5 h is shown in Figure 4, to avoid overlapping of the graphs.

The MWCO rejection rates for the coated membrane with four filtration double layers of PDADMAC/PSS determined at different filtration time intervals are shown in Table 4. Similar rejection rates were observed over all filtration intervals.

The MWCO for the LbL-coated Multibore^®^ membranes was found to be 385 Da, in contrast to 100 kDa for the pristine ultrafiltration membrane. Consequently, the LbL modification was successful.

The zeta-potential for a corresponding flat sheet pristine PES membrane was measured to be ~50 mV at pH 7. Since the PES material is slightly hydrophobic, the negatively measured zeta-potential is primarily attributed to the adsorption of the OH^−^ ions from the medium. On the other hand, the surface charge for the modified membranes was determined via measurement of the zeta-potential of the analogously coated model PES microparticles, as described in Section 2.3. The measurement showed that the surface charge of the microparticles coated with the PSS layer is negative (−40 mV), while the zeta-potential of the microparticles coated with the PDADMAC layer is positive (+40 mV). Accordingly, the polyanion and polycation coatings shifted the surface potential either to a negative or positive surface charge, respectively. The final filtration layer, consisting of four double layers of PDADMAC/PSS, is negatively charged (−40 mV). The negative surface charge is expected to contribute to the repelling of the negatively charged DNA. A positive shift in net charge, which has been described for coatings with a higher amount of bilayers [45], could not be observed here.

### 3.2. Pure Water Permeability and Salt Retention

**Figure 5:**
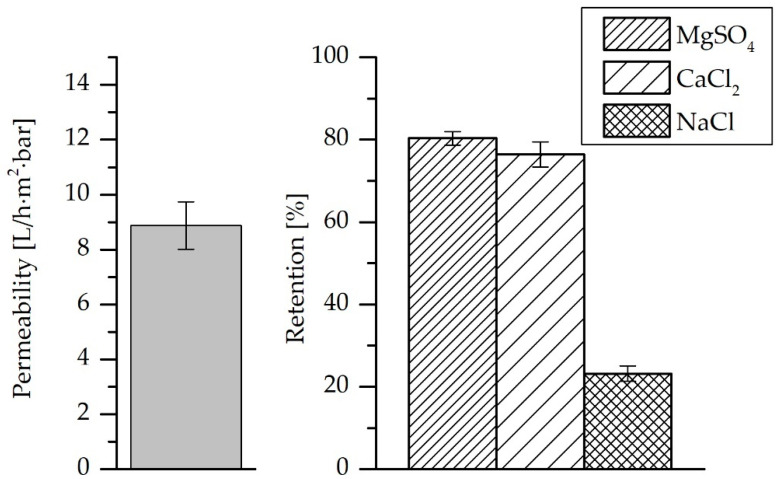
Measurement of pure water permeability and salt retention for LbL-coated membranes.

The LbL-coated membrane exhibited an average pure water permeability of 8.9 ± 0.9 L·h^−1^·m^−2^·bar^−1^. Besides, the salt retention of MgSO_4_, CaCl_2_ and NaCl were plotted in Figure 5. It is worth mentioning that the pristine membrane is an ultrafiltration membrane; therefore, it is obvious that no salt retention tendency should be expected and/or measured. The LbL-coated membranes showed MgSO_4_ retention of almost 80% and CaCl_2_ retention of 76%. On the other hand, the retention for NaCl was 23%. The retention of monovalent salts is significantly lower than that of divalent salts. The negative charges on the LbL-coated membranes, as illustrated by the zeta-potential measurement, improved the retention of the SO_4_^2−^ ions and attracted the divalent cations more strongly, even though their entry was restricted by exclusion effects. Moreover, bigger SO_4_^2−^ ions than void openings in the LbL-coating compared to the Cl^−^ ions may explain the better retention of MgSO_4_ in comparison to CaCl_2_, in order to maintain the electroneutrality condition as described in [47]. Accordingly, the retention values can be explained by a combination of size exclusion and Donnan exclusion mechanisms (related to the size and charge of respective ion in single salt solution) [47]. This is in addition to the dielectric exclusion mechanism that is related to the tendency of ions to shed their hydration shell to enter the membrane. Since the dielectric exclusion energy for divalent ions is higher than that for monovalent ions, the dielectric effect at the membrane surface supports a better separation of the divalent ions [48]. Moreover, lower NaCl retention might be explained by the alteration of membrane surface charge via individual ion adsorption phenomenon [49]. Negatively charged LbL-coated membranes might become neutrally charged due to cation adsorption, leading to better retention of CaCl_2_ than NaCl.

### 3.3. ARG Retention

**Figure 6:**
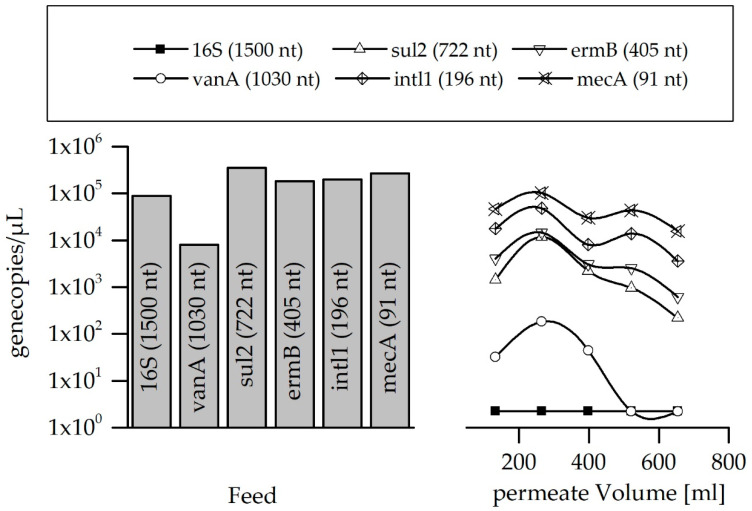
Reduction in gene copy numbers for the uncoated membrane during a filtration of 650 mL in total. Limit of quantification (LOQ) was determined to be 10 gene copies∙µL^−1^.

To determine the effect of the LbL coating, the uncoated (pristine) membrane was also tested on the retention of ARG fragments of different lengths (double-stranded DNA fragments ranging from 91 to 1500 nucleotides). The gene copy concentration of the feed and the permeate are shown in Figure 6.

The concentration of the ARG fragments of different lengths in the permeate fraction increased clearly with decreasing nucleotide number. The largest investigated fragment (16S, 1500 nt) was not detected in the permeate at all. The fragment of 1030 nt (vanA) was completely reduced below the limit of quantification (LOQ) after filtering more than 400 mL. Resistance genes have an average length of 800 to 1200 nt when they are intact and are potentially transferable among bacteria. Therefore, representative genes were selected with similar lengths to investigate whether the ultrafiltration membranes are able to reject the exemplary intact resistance genes. It was shown that ARGs of comparable size can be successfully rejected by the membrane and only smaller fragments can pass the pores. The conventional methods to detect resistance genes with qPCR use only parts of 100–400 nt of the genes for their quantification [50]. During the on-site application of the Multibore^®^ ultrafiltration membranes in water treatment, it can be assumed that if the ARGs are detected in the permeates, this would be only the defect and fragmented genes.

**Figure 7:**
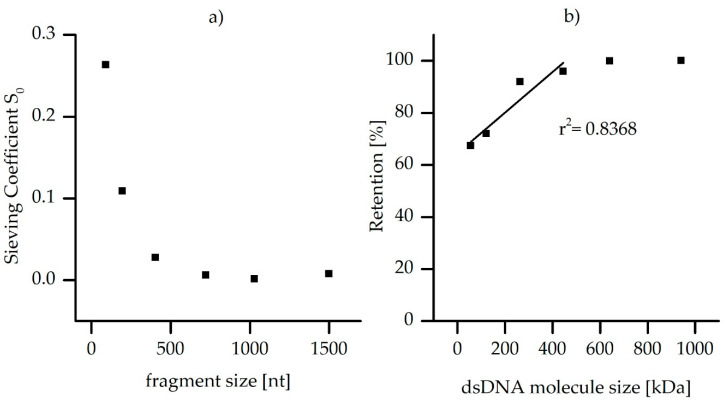
(**a**) The sieving coefficient, S_0_, related to the tested fragment sizes. (**b**) The relative retention depending on the DNA molecule size, to calculate the membrane cutoff for double-stranded DNA.

The sieving coefficient was determined according to Equation (5), dividing the absolute gene copies of the pooled permeate fraction with those in the feed. Figure 7 shows the calculated sieving coefficient depending on the DNA fragment sizes and the retention efficiency for the uncoated (pristine) ultrafiltration membrane related to the molecule sizes of the tested DNA fragments.

The sieving coefficient decreased significantly with fragment size, resulting in an S_0_ of 0.008 and lower for fragments larger than 722 nt. For calculation of the retention depending on molecule size, the second permeate sampling point (after membrane equilibration and lowest fouling progress) of two filtration experiments was chosen (data of the second filtration experiment not shown). A linear regression of the molecule sizes showed significant retention in both independent experiments. A determination of the cutoff based on the regression could not be derived exactly for the uncoated pristine membrane for dsDNA molecules under employed conditions (due to a statistically insignificant number of data points between 100 and 300 kDa). Based on the dsDNA, a 90% retention can be limited between the dsDNA gene fragments of 121 and 263 kDa. Compared to the MWCO of 100 kDa, previously determined using with PEG/PEO mixture, this value is slightly higher. It is presumed that the super helical structure of the DNA molecules is stretched out by shear in the pores, which was also concluded in a previous study investigating UF membrane penetration by a 9.5 kbp plasmid [51]. In this study, the observations revealed that even the tested plasmid of 5871 kDa was able to penetrate the pores of a 20 kDa PES membrane, obviously independent of the electrostatic repulsion between the strongly negatively charged DNA and the negatively charged PES membrane (cf. Section 3.1). In another study, also dealing with the permeation of DNA molecules through ultra- and nanofiltration pores, similar effects can be observed. Another work also gave the conclusion that linearized DNA has a higher elongation and flexibility [52]. This means that it can be more easily captured by the flow field during pore filtration, and thus it passes the membrane more easily. Therefore, it is not surprising that also ARG fragments of 121 to 263 kDa could penetrate the uncoated UF membranes during the dead-end filtration. In comparison to the results by Arkhangelsky and Reif [51,52], where bigger DNA molecules (than the ones tested in the current study) were insufficiently rejected by UF membranes, it could be shown here that ARG fragments larger than 263 kDa were rejected by the uncoated membrane.

**Figure 8:**
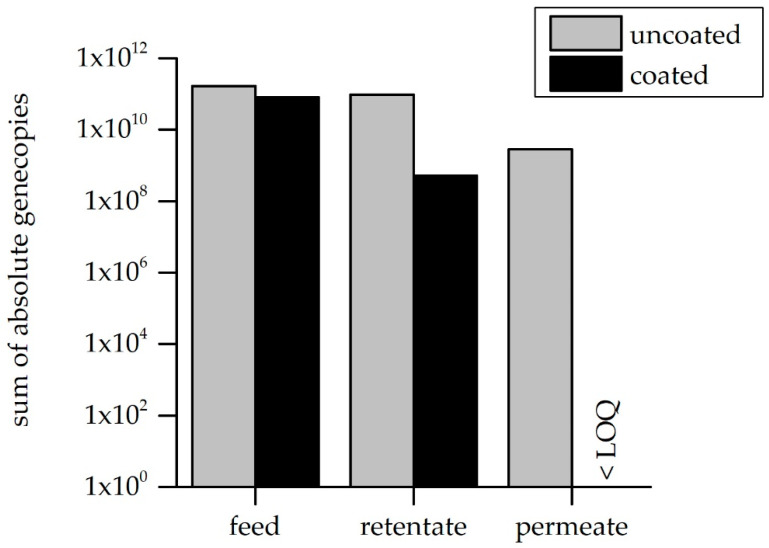
Sum of the absolute gene copy numbers for all tested fragments in the feed, retentate and permeate fraction for the uncoated (pristine) module, compared to the LbL-coated membrane. The LOQ (limit of quantification) is 10 gc/reaction (with a 1 µL sample).

In all the performed experiments, it could be clearly observed that coating with four filtrating double layers of PDADMAC/PSS resulted in the complete retention of all the gene fragments, even for the smallest fragment (91 nt). In Figure 8, the absolute gene copy numbers per fraction, summed up for all the tested fragments, is shown.

**Figure 9:**
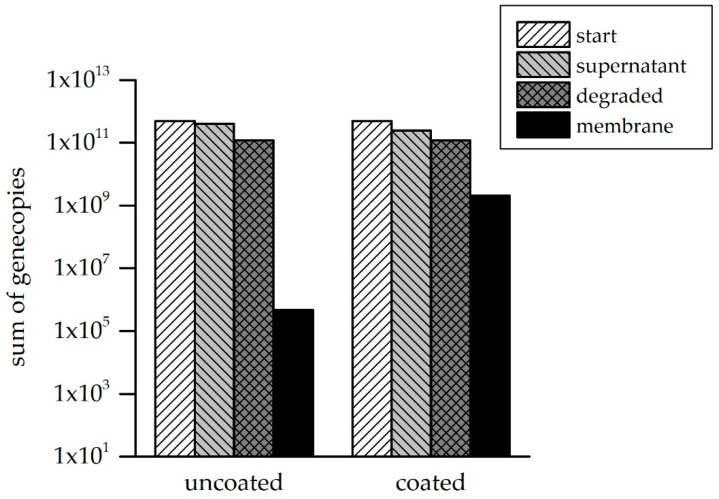
Sum of the absolute gene copy numbers quantified in the supernatant on the membrane and in the supernatant after 24 h in the experimental approach without a membrane (negative control), which was used to calculate the degradation of the gene fragments over the 24 h time period. The mean start value for all four incubation experiments with very low standard deviation (negative control, uncoated and coated) is shown here.

The overall retention efficiency of 2 log levels for the uncoated membrane, which equates to a 98.3% removal of the filtered ARG fragments, is optimized to complete the retention for the coated module. The measured gene copy number in the retentate of the uncoated membrane approximately equals the number calculated by subtracting the permeate number from the feed. No gene fragments could be quantified in the permeate of the coated membrane, and thus the permeate gene copy number is below the number in the feed fraction. These results are consistent with the measured MWCO (i.e., 384 Da). Nonetheless, not only the lower MWCO of the coated membranes seems to lead to complete ARG rejection, but also interactions between the DNA and LbL-coating are assumed to be slightly higher compared to the uncoated membrane. Therefore, non-filtration tests were carried out to determine whether the coating with layers of polyanions and polycations may chemically or physically interact with the ARG fragments. In the incubation experiments, described in Section 2.6, the two membrane types (LbL-coated and pristine) were cut equally and incubated in ARG-spiked sterile ultrapure water for 24 h. With sampling of the working solution at t = 0 h and t = 24 h, as well as DNA extraction from the membranes, the ARG/membrane interaction could be determined. Figure 9 shows the results for all investigated fractions.

The amount of gene copies in the supernatant is similar for the uncoated and the coated fragment. The absolute number of degraded gene fragments could be calculated by the detected reduction of the gene fragments over a 24-h time period in the negative control with the ARG solution but without a membrane. There was a difference detectable in the gene copy numbers determined on the membrane itself, indicating better adsorption of the ARG fragments to the LbL-coated membrane module. Nevertheless, the percentage of the ARG amount adsorbed to the membrane is very low for both modules (0.4% for the uncoated, and 0.0001% for the coated). The membrane coating was designed to not only achieve higher rejection due to the smaller pore size, but also to adjust the surface properties in order to optimize the rejection of organic molecules. The outer layer is negatively charged but a blending of the polycation and polyanion layers always occurs, leading to additional positive charges on the membrane surface and providing a few binding sites for DNA.

### 3.4. Mechnical Stability of the LbL Modification

**Figure 10:**
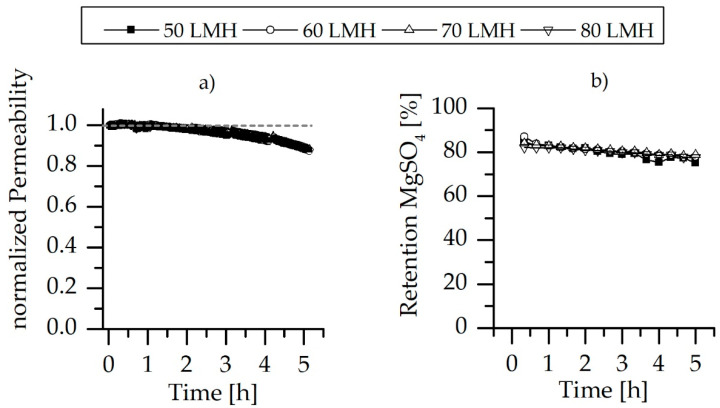
(**a**) Normalized permeability performance during five filtration cycles of a MgSO_4_ salt solution employing different backwashing fluxes, ranging from 50–80 LMH; and (**b**) the MgSO_4_ retention rate measured after the BW experiments.

The stability of the LbL membrane modification was investigated via applying different backwashing fluxes over five consecutive filtration cycles, and the coating stability was tested using MgSO_4_ salt retention. The results are shown in Figure 10. The results indicate that LbL modification was able to withstand the applied backwashing fluxes. The normalized permeability performance, as illustrated in Figure 10a, showed a comparable trend throughout the five filtration cycles during different the BW fluxes experiments; permeability declines of 1%, 2%, 5%, 8% and 13% after the first, second, third, fourth and fifth cycle were observed, respectively. The higher permeability decline at the last filtration cycles may be attributed to the increment of the feed concentration during filtration by a concentration factor of almost 1.15 as a result of the recirculation of the retentate back to the feed tank, while discharging the permeate. In Figure 10b, the LbL-coated membranes showed MgSO_4_ retention of ~80% throughout the five filtration cycles (after mechanical backwashing), independent of the increase in BW fluxes from 50–80 LMH. However, a slight reduction in MgSO_4_ retention was observed in the case of 50 LMH at the end of the fourth filtration cycle, which is mainly attributed to filtration unit instability rather than LbL modification stability.

### 3.5. Chemical Stability

Chemical stability of the LbL-modified membrane was tested by direct exposure to acidic conditions (sulphuric acid at pH 1.5) and basic/oxidizing conditions (50 ppm NaOCl in 100 mM NaOH pH 12). Permeability and salt retention were measured before and after 30 min exposure to the chemicals, which is a typical procedure for regeneration of polymeric membranes.

**Figure 11:**
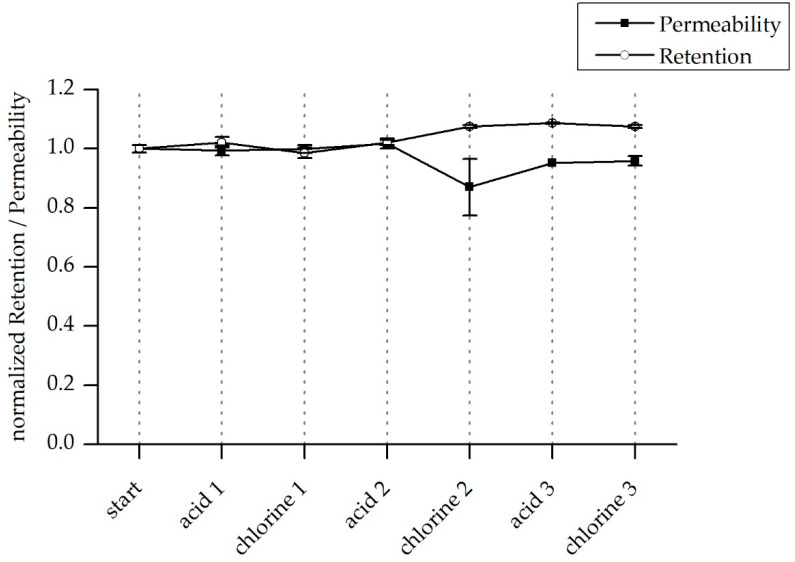
Permeability (black rectangles) and salt retention (white circles) before and after chemical treatment of the LbL-coated membrane.

In general, the results in Figure 11 showed that after three regeneration cycles with acid and chlorine, neither the permeability nor the retention was significantly decreasing. Nevertheless, after two cycles, there was a limited increase in membrane retention and a small decline in permeability compared to the conditions before chemical regeneration. This may be caused by a compaction of the layers during the filtration time; however, no damage for the LbL-coating by either acidic conditions or chlorine treatment was found.

## 4. Conclusions

Multibore^®^ ultrafiltration membranes were successfully modified using polyelectrolyte multilayers. Advanced rejection capabilities towards salts and antibiotic resistance genes (ARGs) were found for the LbL-modified membranes. LbL-coated membranes were characterized by negative surface charges and an MWCO < 400 Da to match the typical characteristics of NF membranes. The membranes modified with four filtrating double layers of PDADMAC/PSS exhibited a pure water permeability of 9 L·m^−2^·h^−1^·bar^−1^ and 80% retention for MgSO_4_ and CaCl_2_, while the NaCl retention was in the range of 25%. Besides, the ARG retention was 100%, such that no gene copies could be detected in the permeate samples. Therefore, the developed LbL-coated membranes are concluded to be able to remove ARGs in water below the detection limit of qPCR. Moreover, the LbL-modified membranes showed very good stability while applying backwashing fluxes in a range of 50–80 LMH. The polyelectrolyte multilayers also withstand chemical cleaning in sulfuric acid at pH 1.5 and in 50 ppm NaOCl at pH 12. Nevertheless, further long-term salt retention filtration and mechanical backwashing experiments will be performed to examine the performance of the newly developed membranes at realistic application conditions. Furthermore, pilot filtration experiments of contaminated surface water with ARGs are planned, to investigate the applicability of the newly modified capillary membranes at full-scale conditions.

## Figures and Tables

**Table 1:** Composition of the feed solution mixture containing PEG of different molecular weights for determination of the MWCO.

PEG Molecular Weight (Da)	Concentration (g·L^−1^)
62	0.52
106	0.39
150	0.34
200	0.30
300	0.25
400	0.22
600	0.18
1000	0.30
1500	0.24
2000	0.22

**Table 2:** Primer sets and resulting fragment size in nucleotides (nt) for the preparation of a gene fragment solution for the filtration experiments.

Gene	Fragment Size (nt)	Forward Primer (5′-3′)	Reverse Primer (5′-3′)	Reference
*16S rDNA*	1500	agagtttgatcctggctcag	ggttaccttgttacgactt	[39]
*vanA*	1030	catgaatagaataaaagttgcaata	cccctttaacgctaatacgatcaa	[40]
*sul2*	722	cggcatcgtcaacataacc	gtgtgcggatgaagtcag	[41]
*ermB*	405	catttaacgacgaaactggc	ggaacatctgtggtatggcg	[42]
*intl1*	196	gccttgatgttacccgagag	gatcggtcgaatgcgtgt	[43]
*mecA*	91	cgcaacgttcaatttaattttgttaa	tggtctttctgcattcctgga	[44]

**Table 3:** Annealing temperatures and elongation times for each qPCR reaction.

Gene	Amplicon (nt)	TA (°C)	tE (s)
*16S rDNA*	1500	58	50
*vanA*	1030	60	35
*sul2*	722	65	25
*ermB*	405	63	20
*intl1*	196	63	20
*mecA*	91	63	20

**Table 4:** Molecular weight cut-off rejection rates for the LbL-coated PES Multibore^®^ measured at three different filtration intervals: 3.5, 4.5 and 5.5 h.

Permeate Collection Time Interval (h)	Rejection Rate at 90%
3.5	383 Da
4.5	385 Da
5.5	384 Da
